# Determining the stability of minimally displaced lateral humeral condyle fractures in children: ultrasound is better than arthrography

**DOI:** 10.1186/s13018-021-02854-z

**Published:** 2021-12-16

**Authors:** Xing Wu, Xiantao Shen

**Affiliations:** Department of Pediatric Orthopedic Surgery, Wuhan Children’s Hospital (Wuhan Maternal and Child Healthcare Hospital); Tongji Medical College, Huazhong University of Science & Technology, 100 Xianggang road, Wuhan, 430016 People’s Republic of China

**Keywords:** Ultrasound, Arthrography, Lateral condyle fractures

**Dear Editor**,

On behalf of my colleagues, I wanted to thank Dr. Rehm et al. for their interest in the findings from one of our recent studies of “Determining the stability of minimally displaced lateral humeral condyle fractures in children: ultrasound is better than arthrography.” I appreciate their comments that support the conclusion of our study. As already mentioned in our publication and emphasized in their comments, the main goals of our research were to introduce an ultrasound technique that would enable determining fracture stability in a simplified manner in order to make it available and usable by orthopaedic surgeons anywhere and not only in special centers.

Since 2001, Vocke-Hell et al. [1] first found that ultrasound can show the fracture line extends through the articular cartilage in lateral humeral condyle fractures (LHCFs). However, we would like to ask why there are so few reports in the literature. In our hospital, ultrasound has been applied to diagnose LHCFs since 2013, and the results of the study have been published [2, 3]. We recommended the routine use of the ultrasound technique. Nevertheless, it is difficult to enable its application in clinical practice in other hospitals. Orthopaedic surgeons are familiar with radiographs but have a poor understanding of ultrasound images. Arthrography is mainly used to assess the integrity of the articular cartilage in LHCFs. In the classification systems recently proposed by Weiss’s study [4], the integrity of the articular cartilage surface was mainly determined on the basis of arthrography. Arthrography is also used to determine the integrity of the cartilage surfaces in China. Therefore, we only used arthrography as a control group for this case–control study. We did not perform arthrography routinely to evaluate these fractures. We commended arthrography is difficult to assess complex three-dimensional articular cartilage of elbow fractures. On the basis of our data, we believe that ultrasound can provide more accurate information than arthrography to determine the statuses of articular surface.

As previously reported in the literature, whether minimally displaced LHCFs should be treated with non-operative treatment, closed reduction and percutaneous pinning (CRPP), or open reduction and internal fixation is controversial. Similarly, we cannot draw conclusions Weiss type II fractures could have been treated safely without CRPP. In the present study, patients diagnosed with a minimally displaced LHCF (2–5 mm) underwent CRPP under general anaesthesia. Besides, if the cartilage hinge was intact, we have also attempted to treat it with a long-arm cast. Therefore, this is a worthy topic that is being studied and the results will be published in the future.

## Data Availability

Not applicable.

## Abbreviations

LHCFs: Lateral humeral condyle fractures
CRPP: Closed reduction and percutaneous pinning
